# A novel dic (17;18) (p13.1;q11.2) with loss of TP53 and BCR/ABL rearrangement in an Imatinib resistant chronic myeloid leukemia

**DOI:** 10.1186/1755-8166-5-36

**Published:** 2012-08-20

**Authors:** Walid Al-achkar, Abdulsamad Wafa, Faten Moassass, Moneeb Abdullah Kassem Othman

**Affiliations:** 1 Molecular Biology and Biotechnology Department, Human Genetics Division, Atomic Energy Commission, Damascus, Syria; 2 Jena University Hospital, Institute of Human Genetics, Jena, Germany; 3 Molecular Biology and Biotechnology Department, Human Genetics Division, Atomic Energy Commission of Syria, P.O. Box 6091, Damascus, Syria

**Keywords:** Dic (17;18), Chronic myeloid leukemia (CML), TP53 gene, T315I, Fluorescence in situ hybridization (FISH), Reverse transcription polymerase chain reaction (RT-PCR), Imatinib resistant

## Abstract

**Background:**

The so-called Philadelphia (Ph) chromosome is present in more than 90% of chronic myeloid leukemia (CML) cases. It results in juxtaposition of the 5′ part of the BCR gene on chromosome 22 to the 3′ part of the ABL gene on chromosome 9. Since the majority of CML cases are currently treated with Imatinib, variant rearrangements in general have no specific prognostic significance, although the mechanisms involved in resistance to therapy have yet to be investigated. The T315I mutation within the abl-gene is the most frequent one associated with resistance to tyrosine kinase inhibitors.

**Results:**

This study evaluated a Ph chromosome positive CML case resistant to imatinib mesylate. A dic(17;18), loss of TP53 gene, co-expression of b2a2 and b3a2 fusions transcript and a T315I mutation were found.

**Conclusions:**

We reported here a novel case of a Ph chromosome positive CML with a secondary abnormality [dic(17;18)], resulting to Glivec resistance but good response to nilotinib. The dic(17;18) might be a marker for poor prognosis in CML. Our finding indicated for an aggressive progression of the disease. The patient died under the treatment due to unknown reasons.

## Background

Chronic myeloid leukemia (CML) is a clonal malignant disorder of a pluripotent hematopoetic stem cell characterized by the presence of the Philadelphia (Ph) chromosome in more than 90% of patients. The Ph chromosome is a product of the reciprocal translocation t(9;22)(q34;q11), which transposes the 3′ portion of the ABL oncogene from 9q34 to the 5′ portion of the BCR gene on 22q11.2. The crucial pathogenetic consequence of this translocation is the creation of a chimeric BCR/ABL gene on the derivative chromosome 22 [1]. The expression of the BCR/ABL chimeric protein with an increased tyrosine kinase activity plays an essential role in the pathogenesis of CML [2]. The progression of CML from chronic phase (CP) to blast crisis (BC) is frequently associated with nonrandom secondary chromosomal aberrations such as +8, i(17q), +19 and an extra Ph chromosome [3]. At the molecular level, mutation of the tumor suppressor gene TP53 located at 17p13 is detected in 25–30% of CML-BC. However, no mutation of the remaining TP53 allele in CML cases with i(17q) has been noted [4].

Knowledge of the biology of CML has enabled targeted therapies in preclinical and clinical oncology. Imatinib (Glivec, formerly STI571) was the first available BCR/ABL targeted therapy and produced complete cytogenetic responses in 70–85% of patients with CML in early CP [5]. However, despite the stunning efficacy of this agent, resistance or intolerance to imatinib can be observed. Moreover, imatinib does not completely eradicate residual leukemic stem cells and progenitors [6,7]. Also, failure to respond to imatinib was in some CML patients result of mutations arising in the BCR-ABL kinase domain (KD), leading to shortened survivals of CML patients with these mutations [8].

T315I is one of the most frequent mutations associated with resistance to tyrosine kinase inhibitors (TKI), not only to the 1^st^ generation TKI such as imatinib, but also to the newly approved 2^nd^ generation TKI such as nilotinib and dasatinib [9].

Here we reported a novel case of a Ph chromosome positive CML with dic(17;18), loss of TP53 gene, co-expression of b2a2 and b3a2 fusions transcript and T315I mutation resulting in Glivec resistance, while good response in nilotinib was observed; i.e. the clone with the dicentric chromosome decreased under this treatment from 100% to 80%.

### Case report

A 19-year old woman was diagnosed with CML in chronic phase (CP) in August 2003 as she had higher white blood cell (WBC) counts and splenomegaly previously. In March 2010, the patient presented for the fifth time (for further details see Table 1) with a WBC of 2.2×10^9^/l consisting of 25% neutrophils, 73% lymphocytes, 1% monocytes and 1% eosinophiles. The platelets count was 111×10^9^/l and the hemoglobin level was 9.5 g/dl. The patient was treated with nilotinib at 800 mg/day for overall 5 months. In November 2010, she passed away under the treatment due to unknown reasons.

**Table 1:** Clinical history of the patient together with diagnostic results and treatment

**Visit No.**	**Date**	**Methods**	**Hematologic parameters**	**Treatment**	**Results**
1	August 2003	GTG, FISH (WCP probes)	WBC 327×10^9^/l with 73% neutrophils, 23% lymphocytes, 3% monocytes and 1% eosinophiles. Hgb 11.9 g/dl and Plts 540×10^9^/l.	-	46,XX,t(9;22)[20]
2	November 2005	GTG, FISH (BCR/ABL and WCP probes)	WBC 7.9×10^9^/l with 66% neutrophils, 31% lymphocytes, 2% monocytes and 1% eosinophiles. Hgb 11.2 g/dl and Plts 371×10^9^/l.	Imatinib mesylate at 200 mg/day for overall 12 months	46,XX,t(9;22)[20]
3	November 2006	GTG, FISH (BCR/ABL probe)	WBC 6.8×10^9^/l with 64% neutrophils, 33% lymphocytes, 2% monocytes and 1% eosinophiles. Hgb 12.8 g/dl and Plts 305×10^9^/l.	Imatinib at 400 mg/day for overall 12 months	46,XX,t(9;22)[20]
4	November 2008; patients interrupted treatment for ~12 months; Nitolib was initiated November 2009	GTG, FISH, RT-PCR, RFLP	WBC 15.5×10^9^/l with 54% neutrophils, 43% lymphocytes, 3% monocytes and 1% eosinophiles. Hgb 8.8 g/dl and Plts 215×10^9^/l.	Imatinib at 400 mg/day for overall 12 months in the total.	45,XX,t(9;22),+dic(17;18),-17,-18[20]
					b2a2 transcript
					T315I mutation
5	March 2010	GTG, FISH, RT-PCR, RFLP	WBC 2.2×10^9^/l with 25% neutrophils, 73% lymphocytes, 1% monocytes and 1% eosinophiles. Hgb 9.5 g/dl and Plts 111x10^9^/l.	nilotinib at 800 mg/day for overall 5 months.	45,XX,t(9;22),+dic(17;18),-17,-18 [16]/46,XX,t(9;22)[4]
					co-expression of b2a2 and b3a2 transcript.
					T315I mutation
6	November 2010	The patient passed away under the treatment due to unknown reasons			

**Figure 1:**
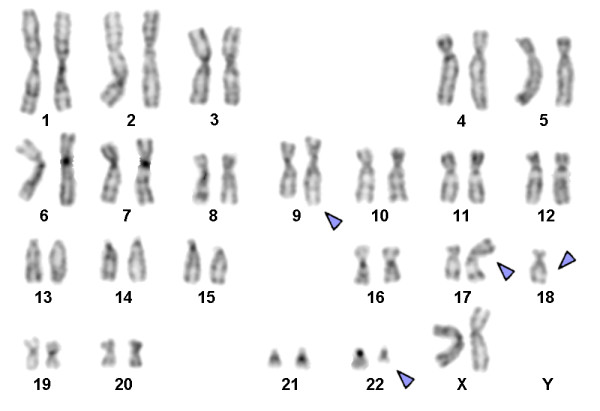
**GTG-banding revealed a complex karyotype with two further aberrant chromosomes besides chromosomes 9 and 22.** All derivative chromosomes are highlighted by arrow heads.

**Figure 2:**
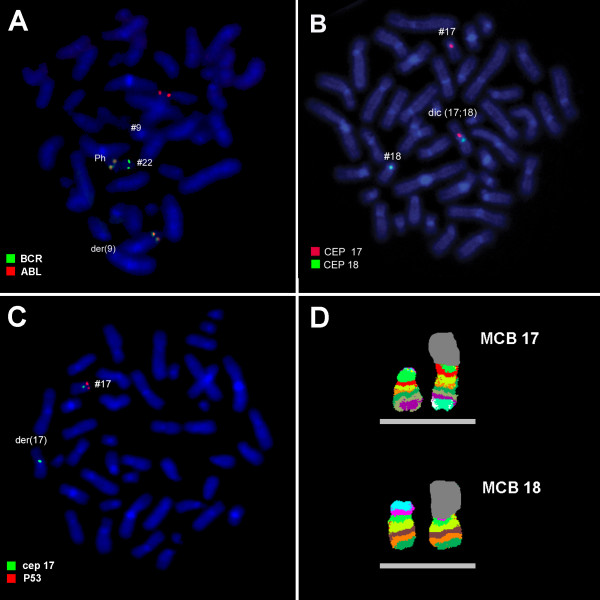
**Karyotype and chromosomal aberrations were confirmed using molecular cytogenetic approaches.** (**A**) FISH using probes for BCR (green) and ABL (red) confirmed Ph chromosome presence. (**B**) FISH with CEP 17 (red) and CEP18 (green) showed the presence of both centromeres on the derivative chromosome in question, indicating a dic(17;18). (**C**) The deletion of TP53 of der(17)(p13.1) was identified using 17p13 (p53) together with a CEP 17 probe. (**D**) The application of aMCB 17 and 18 characterized the dic(17;18)(p13.1;q11.2) comprehensively . Abbreviations: # = chromosome; der = derivative chromosome; Ph = Philadelphia-chromosome.

**Figure 3:**
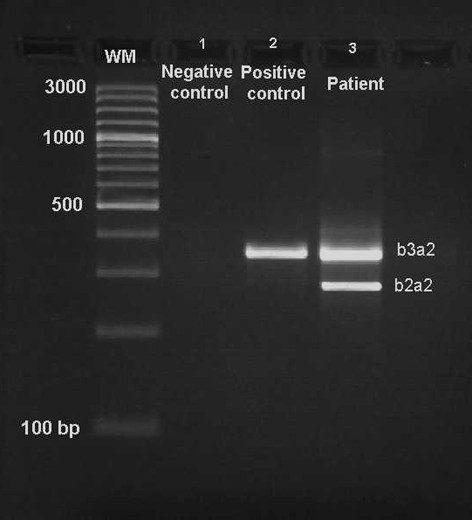
**Gel electrophoresis of the nested RT-PCR products.** Line M, 100 bp molecular weight marker; line 1, negative control; line 2, positive control (b3a2) 353 bp and line 3, coexpression of b2a2 (104 bp) and b3a2 (353 bp) from the patient.

Karyotyping was performed before and after of chemotherapy treatment. The result after chemotherapy (for further details see Table 1) was 45,XX,t(9;22),+dic(17;18),-17,-18 [16]/46,XX,t(9;22)[4] (Figure 1) and further specified by molecular cytogenetic studies (Figure 2). Dual-color-FISH using a probe specific for BCR and ABL revealed that a typical Ph chromosome with BCR/ABL-translocation was present in all studied metaphases and nuclei (Figure 2A). CEP 17 and 18 probes showed a dicentric chromosome leading to deletions of parts of the short arms of the involved chromosomes (Figure 2B). The locus-specific probe 17p13 (p53) confirmed the absence of the 17p on the dicentric chromosome (Figure 2C). Finally, aMCB using probes for the corresponding chromosomes was performed as previously reported [10]; Figure 2D). Thus, the following final karyotype was determined: 45,XX,t(9;22)(q34;q11),+dic(17;18)(p13.1;q11.2),-17,-18[16]/46,XX,t(9;22)(q34;q11)[4]. The RT-PCR demonstrated co-expression of b2a2 and b3a2 fusions transcripts as most often found in CML (Figure 3).

**Figure 4:**
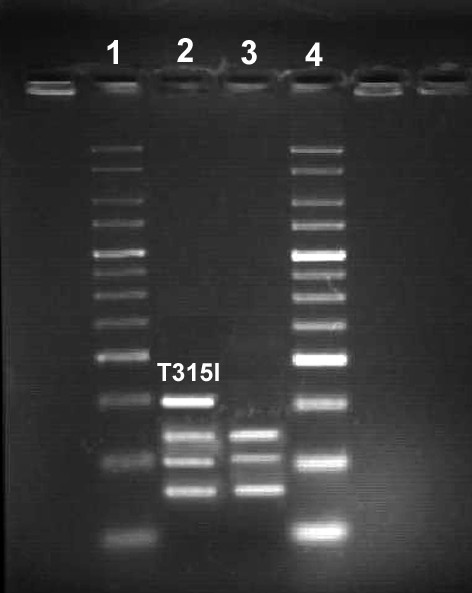
**Gel electrophoresis of the DdeI restriction analysis on the ABL exon 7 and progressive appearance of the T315I point mutation.** A single base change from C to T results in a fragment length polymorphism. The T315I mutation resulted in an uncut PCR product of 72 bps. Lines 1 and 4, 25 bp molecular weight markers; line 2, T315I mutation from the patient and line 3, normal control (K562 cell line).

The typical T315I mutation was detected by DdeI restriction enzyme digestions, i.e. a single base pair transition (C to T) resulted in a restriction fragment length polymorphism (Figure 4).

## Discussion

According to the literature, a dic(17;18) is a recurrent cytogenetic abnormality in acute myeloid leukemia (AML) [dic(17;18)(p12;p11) [11]], chronic lymphocytic leukemia (CLL) [dic(17;18)(p11.2;p11.2) [12]] and CML-BC [dic(17;18)(p11;p11) [13]]. To the best of our knowledge, the present case is the only ever seen case of Ph chromosome-positive CML-CP with dic(17;18)(p13.1;q11.2), loss of TP53 gene, co-expression of b2a2 and b3a2 fusions transcript and T315I mutation resulting in Glivec resistance [14].

During CML progression, isochromosome (17)(q10) is one of the non-random changes. This aberration is associated with loss of TP53 and mostly with poor prognosis [15]. Point mutation and/or deletion of the TP53 gene are regarded as potentially important steps in the development of various hematological malignances, low response to chemotherapy, and short survival [16,17]. The dic(17;18) led also to the loss of p53 gene.

Most of the CML patients express b2a2 or b3a2 of BCR-ABL mRNA encoding for p210 Bcr-Abl tyrosine kinase [18]. In this study we found co-expression of b2a2 and b3a2. Co-expression of more than one type of fusion transcript in a patient may be caused by alternative splicing or phenotypic variation, with clinical courses different from classic CML [19].

A point mutation in the ABL part of the ABL-BCR fusion protein resulting in a T315I change can be found in CML patients which are resistant to Glivec. It has been reported that 50–90% of hematological relapse is associated with an ABL point mutation in the ATP binding site, and the catalytic domain or the activation loop of the ABL kinase domain [20]. Molecularly, mutations have been frequently found involving the kinase domain of the BCR-ABL gene. Particularly poor prognoses are associated with mutations in the ATP loop [21]. Dasatinib is effective against most mutants of ABL in vitro and in Imatinib-resistant CML patients [22]. Most of the patients with BCR-ABL mutations achieved a clinical response under dasatinib, even in mutations not explored in vitro. However, despite high impact in vitro and even if all patients showed improvement of their clinical state, various levels of responses are seen in the patients [22].

In the present case, five months after the start of treatment with nilotinib, GTG and FISH showed absence of dic(17;18) in 20% of the cells but a remainder Ph chromosome in 100% of metaphases studied.

In conclusion, we reported here a novel case of a Ph chromosome positive CML with dic(17;18), loss of TP53 gene, co-expression of b2a2 and b3a2 fusions transcript and T315I mutation resulting to Glivec resistance but good response to nilotinib. The dic(17;18) might be a marker for poor prognosis in CML.

## Materials and Methods

### Chromosome analysis

Chromosome analysis using GTG-banding was done according to standard procedures before and after chemotherapeutic treatment [23]. A total of 20 metaphase cells derived from unstimulated bone marrow culture were analyzed. Karyotypes were described according to the International System for Human Cytogenetic Nomenclature [24].

### Molecular cytogenetics

Fluorescence in situ hybridization (FISH) using LSI BCR/ABL dual color dual fusion translocation probe (Abbott molecular/Vysis, USA), centromere-specific probes (CEP) for chromosomes 17 and 18 (Abbott molecular/Vysis, USA) were applied according to manufacturer’s instructions together with 17p13 (p53) (Q-Biogene, USA) [25]. Array-proven multicolor banding probe (aMCB) sets based on microdissection derived region-specific libraries for chromosome 17 and 18 were applied as described [10]. A total of 20 metaphase spreads were analyzed, each using a fluorescence microscope (AxioImager.Z1 mot, Zeiss) equipped with appropriate filter sets to discriminate between a maximum of five fluorochromes and the counterstain DAPI (4′,6-diamino-2-phenylindole). Image capturing and processing were carried out using an ISIS imaging system (MetaSystems, Altlussheim, Germany).

### Reverse transcriptase-polymerase chain reaction (RT-PCR) for BCR/ABL fusion transcripts

RT-PCR was carried out as previously described [26].

### Restriction fragment length polymorphism (RFLP) analysis

RFLP analysis was performed as previously described [20].

## Consent

Written informed consent was obtained from the patient for publication of this case report and accompanying images. A copy of the written consent is available for review by the Editor-in-Chief of this journal.

## Competing interests

The authors declare that they have no competing interests.

## Authors’ contributions

AW and FM performed the cytogenetic studies in the present case and collected the data relative to this case report. WA supervised the cytogenetic analysis as Director of the MBBD HGD. AW, FM and MAKO did the molecular cytogenetic analysis and interpretation. AW drafted the paper and all authors contributed to the finalizing of the manuscript. All authors read and approved the final manuscript.
